# Mr. Simmons, on Digitalis

**Published:** 1801-08

**Authors:** W. Simmons

**Affiliations:** Manchester


### *To the Editors of the Medical and Phyſical Journal.*

GENTLEMEN,

MUCH as the medical properties of Digitalis have been lately inveſtigated, it has been little employed in ſurgery. Yet from my own experience of its powers, it is, I think, greatly entitled to notice, and ſecondary to none of the more uſeful remedies except bark, opium, and mercury. At preſent, however, I ſhall confine myſelf to the ſtrangulated hernia; and as a proof of its efficacy in it, ſhall ſubjoin the following caſe.

On the 19th of June my aſſiſtance was requeſted for a young man labouring under an incarcerated ſcrotal hernia on the right ſide, for which he had formerly worn a truſs. The deſcent was occaſioned by over-exertion at his buſineſs as a weaver, and was attended with the uſual ſymptoms. Before I ſaw

ſaw him, reduction had been attempted, after employing phlebotomy and calomel, the warm bath, enema nicotianæ, and a ſolution of ſal ammoniac locally; and as inflammation had ſupervened in the ſcrotum on the ſame ſide, leeches had been applied to it. The failure of theſe means preſented the operation by inciſion as a final reſource; yet, not entirely diſcouraged by diſappointment, the enema nicotianæ was ordered to be repeated, ice to be applied to the ſcrotum and frequently renewed, and a grain of Digitalis to be immediately adminiſtered. In two hours their ſedative effects were ſtrongly manifeſted, all reſiſtance was ſubdued, and the hernia now reduced.

I have aſcribed the chief merit to the Digitalis, becauſe the enema nicotianæ and cold applications had previouſly failed, and the circulation was interrupted in a remarkable degree. The pulſe was hardly perceptible, the countenance ſunk, and the general depreſſion ſo great, that æther and other ſtrong ſtimulants were judged expedient after the return of the inteſtine.

Foreign writers have inſiſted much on reſorting early to inciſion, which, though dexterouſly performed, is certainly hazardous. But I am under a perſuaſion, that the neceſſity for it may be ſuperſeded, in general, by the proper employment of theſe ſedatives. The exceptions to which I allude are, where from the particular manner of deſcent, the efforts at reduction oppoſe an obſtacle to the return of the inteſtine; a variety of which I have ſeen, and which muſt baffle every other reſource of art: Otherwiſe, very few caſes, I apprehend, would prove irreducible. Where a ſingle doſe of the Digitalis does not anſwer, it may be repeated every ſecond or third hour, in conjunction with the means commonly employed, according to their ſenſible effect; relying on the judgment of the practitioner in attendance when to abandon them. And I muſt caution him not to continue the ice too long, eſpecially when mixed with ſalt, by which a greater degree of cold is generated, leſt gangrene ſhould be induced, and the event of the operation, if neceſſary, rendered more doubtful.

Reduction is chiefly prevented by the inflammatory ſtate of the contents of the herniary ſac; which ſtate conſiſts in an increaſed frequency and vigour of the arterial pulſations, producing an effuſion, and accompanied with a morbid ſenſibility and tenſion. Theſe augmented dimenſions are further extended, by the hindrance given to the return of the venous blood, and by the watery fluids of the inteſtines diſtilling into the ſtrangulated cavity, as well as by the expanſion of the gas contained within it. While, on the one hand, every cauſe of dilatation dilatation muſt tend to lighten the ſtricture, admitting the tendon to be wholly paſſive; on the other, a diminution of bulk muſt obviouſly facilitate the replacement of the protruded contents. The ſedatives employed in this caſe ſeem well calculated to fulfil the ſeveral indications. By the Digitalis the ſupply by the arteries is ſtinted, and muſcular reſiſtance obviated by it and the nicotiana; and it is the well known property of cold to diminſh ſenſibility, and contract the circumference of ſubſtances coming in contact with it. I have ſaid nothing of inverting the body, ſuppoſing it to be done in every inſtance attended with difficulty, as in this. My method is, to ſuſpend the patient by the hams over the ſhoulders of a ſtrong perſon, ſitting or kneeling on a bed, and to incline the head and body a little forward; in this poſition, the inteſtines recede towards the diaphragm, aſſiſt by their weight the external force, and draw back firſt the part laſt protruded; the fluids may alſo eſcape into the abdomen by their own gravity, and the muſcles immediately concerned are ſtill further relaxed. Coarſe as it is, the one adopted by our anceſtors was more ſo; but their object was the ſame. It may not be amiſs to remark, that the fluid contained in the incarcerated inteſtine ſhould be diſcharged by preſſing laterally, or by endeavouring to approximate its ſides, and not by preſſing from the fundus upwards. Where the aperture is not very ſtrongly cloſed, after reducing the tenſion, a little time ſpent in this way will ſeldom fail to empty it; for the ſtricture muſt be tight indeed to reſiſt the tranſmiſſion of a fluid thus impelled by the hands of the operator.

You have before recorded an inſtance of the ſucceſs of Digitalis in the ſtrangulated hernia (ſee Vol. iii. p. 330,) of which this may therefore be deemed a confirmation.

I am, &c.

